# SOCIAL DETERMINANTS OF HEALTH AND QUALITY OF LIFE AMONG OLDER ADULTS NEW TO LONG-TERM SERVICES AND SUPPORT

**DOI:** 10.1093/geroni/igac059.1939

**Published:** 2022-12-20

**Authors:** Onome Osokpo, Karen Hirschman, Liming Huang, Mary Naylor

**Affiliations:** University of Pennsylvania, Philadelphia, Pennsylvania, United States; University of Pennsylvania, Philadelphia, Pennsylvania, United States; University of Pennsylvania, Philadelphia, Pennsylvania, United States; University of Pennsylvania - School of Nursing, Philadelphia, Pennsylvania, United States

## Abstract

The number of older adults requiring long-term services and support (LTSS) is increasing. Social determinants of health (SDoH)─underlying economic, social, and structural factors─fundamentally shape people’s health including quality of life (QoL). However, the influences of SDoH factors on QoL are relatively unexplored among older adults new to LTSS. The purpose of this study was to examine the relationship between SDoH factors and overall QoL ratings among older adults new to LTSS using data from a prospective NIA funded longitudinal study of change in health related QoL in this population. QoL was measured using a single item: “How would you rate your overall quality of life at the present time?” (1- Poor to 5- Excellent). SDoH variables included years of education, marital status, food security, spirituality, social isolation, financial resources, and social support measures. A full multivariable regression analysis was performed adjusting for age, sex, cognitive status, functional status, physical and emotional health, and LTSS type. Among the 470 participants, 71% were female, 51% were white, 20% were married, and the average age was 81 years (+/- 8 years). Having adequate financial resources to pay for health care needs (p <.0001) was positively associated with QoL. No other SDoH factors were significant. Access to resources to meet health care needs is critically important in promoting the quality of life of older adults especially at the start of LTSS. Implications for future research and policy will be discussed.

